# Evolution of ageing, costs of reproduction and the fecundity–longevity trade-off in eusocial insects

**DOI:** 10.1098/rspb.2017.0380

**Published:** 2017-07-12

**Authors:** Pierre Blacher, Timothy J. Huggins, Andrew F. G. Bourke

**Affiliations:** School of Biological Sciences, University of East Anglia, Norwich Research Park, Norwich NR4 7TJ, UK

**Keywords:** bee, lifespan, life history, senescence, social insect

## Abstract

Eusocial insects provide special opportunities to elucidate the evolution of ageing as queens have apparently evaded costs of reproduction and reversed the fecundity–longevity trade-off generally observed in non-social organisms. But how reproduction affects longevity in eusocial insects has rarely been tested experimentally. In this study, we took advantage of the reproductive plasticity of workers to test the causal role of reproduction in determining longevity in eusocial insects. Using the eusocial bumblebee *Bombus terrestris*, we found that, in whole colonies, in which workers could freely ‘choose’ whether to become reproductive, workers' level of ovarian activation was significantly positively associated with longevity and ovary-active workers significantly outlived ovary-inactive workers. By contrast, when reproductivity was experimentally induced in randomly selected workers, thereby decoupling it from other traits, workers' level of ovarian activation was significantly negatively associated with longevity and ovary-active workers were significantly less long-lived than ovary-inactive workers. These findings show that workers experience costs of reproduction and suggest that intrinsically high-quality individuals can overcome these costs. They also raise the possibility that eusocial insect queens exhibit condition-dependent longevity and hence call into question whether eusociality entails a truly reversed fecundity–longevity trade-off involving a fundamental remodelling of conserved genetic and endocrine networks underpinning ageing.

## 1. Introduction

The evolution of ageing, costs of reproduction and life history represent central, interlinked topics in evolutionary ecology [1–3]. According to the well-supported evolutionary theory of ageing, random extrinsic mortality through accidents or predation inevitably causes older cohorts to make smaller reproductive contributions to future generations. This weakens natural selection on beneficial alleles in older cohorts and leads to the evolution of the progressive decrease in performance, survivorship or fecundity with time that defines ageing. As selection then seeks to optimize reproductive success rather than longevity, and as limited resources enforce a compromise between investment in reproduction and somatic maintenance, reproduction thereby triggers ageing and entails costs to individuals [1,2]. The result is generally, though not universally [3–6], a trade-off or negative association between fecundity and longevity [2,7].

Following the development of the evolutionary theory of ageing, it has been realized that sociality potentially alters patterns of ageing relative to those found in non-social organisms [8–10]. In this respect, eusocial insects (ants, bees, wasps and termites) represent prominent examples [11–15]. In eusocial insects, queens (specialized reproductive phenotypes) and workers (non-reproductive or non-specialized reproductive phenotypes) differ remarkably in longevity despite arising from the same genomes, with queens in some species outliving workers by a factor of 60-fold [16–18]. Theory suggests that this occurs because the age of first reproduction in queens occurs relatively later than the age of first helping in workers. This difference is then amplified by queens receiving aid from workers and remaining within the protected environment of the nest, while workers experience greater extrinsic mortality through performing risky external tasks such as foraging [8,10,16].

In this way, eusociality leads to highly long-lived queens, which form—by definition—a specialized reproductive caste. For this reason, it has also been suggested that, within the queen caste, the conventional fecundity–longevity trade-off found in non-social organisms has been reversed, leading to a positive association between fecundity and longevity (reviewed in [15]). Evidence for this comes from studies of ants and bumblebees demonstrating a positive association of queen longevity with egg production [19–21] and adult productivity [22]. This positive association does not appear to be simply a function of longer-lived adult queens receiving more resources, as, in *Cardiocondyla* ants, the same pattern is found even when queens are kept in isolation [17]. Moreover, although *Cardiocondyla* queen reproduction appears to be traded-off against an experimentally induced immune response [23], queens did not show reduced longevity when their reproductive effort (egg-laying rate) was experimentally upregulated [24]. This last result suggests that, at the individual level, queens in eusocial insects may not incur conventional costs of reproduction [24]. Such a reversal of the fecundity–longevity trade-off may result from a remodelling of conserved genetic and endocrine networks regulating ageing, reproduction and immunity [25–28].

Positive associations between fecundity and longevity have also been found in reproductive workers in eusocial insects. In some eusocial Hymenoptera (ants, bees and wasps), unmated workers can produce offspring either through arrhenotoky (asexual production of males associated with haplodiploidy) or secondary thelytoky (asexual production of females) [29,30]. Positive correlations between reproduction and longevity have been reported in workers in both arrhenotokous and thelytokous species [31–33]. Reproductive workers might resemble queens in this respect because workers that start reproducing typically refrain from risky external tasks and so remain protected within the nest [34–37].

Despite these suggestive findings, very few studies have tested the hypothesized positive fecundity–longevity association in eusocial insects experimentally [23,24,27,38]. In eusocial insects, attaining reproductive status often depends on intrinsic quality owing to, for example, queen quality affecting survivorship [39] or social regulation mechanisms restraining worker reproduction [40]. Hence, conceivably, individuals that become successful reproductives are a subset of high-quality individuals that can overcome the costs of reproduction. Therefore, the causal role of reproduction in the fecundity–longevity association in eusocial insects remains unclear. Within the queen caste, it is difficult to test this role experimentally by randomizing which individuals become reproductive, because all queens are phenotypically adapted to reproduce. By contrast, such a test is possible within the worker caste as workers in some species are able to switch between reproductive or non-reproductive phenotypes depending on social conditions [41]. In this study, for the first time, we took advantage of the reproductive plasticity of workers to test the causal role of reproduction in determining longevity in eusocial insects.

Our study system was the annual eusocial bumblebee *Bombus terrestris*, in which worker ovarian activation and egg-laying regularly occur, especially after the queen's death, with up to 45% of workers typically laying male eggs in the second half of the colony cycle [40,42]. We performed two experiments. In Experiment 1, we tested for the existence and directionality of the association of worker longevity and reproduction (ovarian activation) in queenright (with a queen) or queenless whole colonies in which workers could freely ‘choose’ whether to become reproductive or not (henceforth termed ‘whole colonies’). This confirmed that, in *B. terrestris* as in other species, individuals that become reproductive in whole colonies live longer than non-reproductive ones. In Experiment 2, exploiting the technique of Alaux *et al*. [41] to induce reproductivity in workers, we tested for the existence and directionality of the association of worker longevity and reproduction when randomly chosen workers are forced to become reproductive. Experimentally decoupling reproduction from intrinsic quality provided evidence that, in fact, workers experience costs of reproduction. The positive fecundity–longevity association in workers in whole colonies is therefore likely to be a function of high-quality workers overcoming these costs, with key implications for our understanding of the influence of sociality on ageing.

## 2. Material and methods

### (a) Colony rearing

For both experiments, colonies of *B. terrestris audax* were obtained from a commercial supplier. Colonies were received a few days after the eclosion of the first workers and each contained the colony queen plus brood. They were transferred to wooden nest-boxes with clear lids and reared under standard conditions, i.e. at 28°C and 60% relative humidity, in constant darkness, and with dried pollen and artificial nectar provided ad libitum. All monitoring, maintenance and marking of the colonies was conducted under red light. For further details of all methods, see the electronic supplementary material.

### (b) Experiment 1: association of worker longevity and ovarian activation in whole colonies

#### (i) Colony set-up and measurement of worker longevity

Three days after receipt of colonies, all adult workers present were individually marked with a numbered and coloured plastic disc glued to the thorax. Thereafter, all colonies were inspected every 1–3 days and all newly eclosed workers present were likewise individually marked and the date was recorded as each worker's date of eclosion. Marking was continued until 74 days after the first worker marking, although it was not continuous in all colonies (see the electronic supplementary material).

During colony inspections, any marked, dead workers were removed, their identity was noted and the date was recorded as the removed worker's date of death. Worker longevity was therefore calculated as the difference between a given worker's recorded dates of eclosion and death. Because inspections were not conducted daily, the true dates of eclosion and death of each worker may have varied from the recorded ones by 1–3 days. All removed workers were frozen at −20°C for later dissection.

To increase the number of workers activating their ovaries, the colony queen was removed from eight colonies randomly selected from the 23 colonies with a queen remaining at day 47 after first worker marking. In addition to two colonies in which the queen had already died naturally, these colonies formed a subsample of 10 queenless colonies. Following queen removals, all colonies were maintained until there were only one to two living workers left per colony, at which point colony rearing was terminated.

#### (ii) Ovary dissections and wing cell measurements

Ovary dissections were conducted on a sample of marked workers for which both eclosion and death dates were recorded. The length of the single longest visible oocyte was measured under a dissection microscope with a software measuring tool, and the same method was used to measure the length of the radial cell in a forewing as an index of the total body size [43]. The length of the longest oocyte was used as a continuous measure of the level of worker ovarian activation, ranging from 0 (no identifiable oocyte present) to 3.4 mm (fully developed egg present). Workers were also classified as ovary-active or inactive according to the presence or absence of identifiable oocytes in their ovaries, respectively. All ovarian dissections and wing cell measurements were performed blindly with respect to worker longevity.

#### (iii) Sample details

Because of possible social and cohort effects on the relationship between worker longevity and level of ovarian activation, worker samples (*n* = 194) were selected on the basis of their having known longevities, falling into the following classes and each having at least one value for oocyte length or wing cell length (see electronic supplementary material for further details). Final sample sizes were as in ‘Results’ and were less than those given here because of additional exclusion of samples as detailed in ‘Statistical analyses’:

(i) Workers both eclosing and dying pre-queen removal/death (*n* = 85 workers from 17 colonies; median (range) = 4 (2–10) workers per colony). These workers, therefore, experienced the queen's constant presence throughout their adult lifetimes.
(ii) Workers both eclosing and dying post-queen removal/death (*n* = 56 workers from six colonies; median (range) = 4 (1–24) workers per colony). These workers, therefore, experienced the queen's constant absence throughout their adult lifetimes.
(iii) Workers eclosing pre-queen removal/death and dying post-queen removal/death (*n* = 53 workers from 17 colonies; median (range) = 3 (1–7) workers per colony). These workers, therefore, experienced a mixed level of the queen's presence over their adult lifetimes, i.e. living partly in her presence and partly in her absence.

### (c) Experiment 2: association of worker longevity and ovarian activation in randomly selected workers

#### (i) Experimental treatments

Upon colony arrival, all newly eclosed workers were individually marked as in Experiment 1 and then reintroduced into their natal colony. After 3 days (to allow social integration), workers from the same age-group (‘focal workers’ hereafter) were taken and assigned to two social treatments at random (by assigning successive workers alternately to the treatments). These treatments (‘F+’ and ‘F−’ treatments) were designed, respectively, to stimulate and inhibit reproductive activity in workers. In both treatments, the focal workers were individually placed in separate plastic boxes with two sister workers (non-focal workers). In the F+ treatment, single 3-day-old focal workers were isolated with two 1-day-old sister workers. In this social context, a dominance hierarchy is established in which the oldest worker (here the focal worker) becomes an egg-layer with fully activated ovaries [41]. In the F− treatment, single 3-day-old focal workers were isolated with two 7-day-old sister workers that had themselves previously been isolated as a pair for the previous 5 days. In this social context, because the two non-focal workers have already established a dominance hierarchy, the focal workers are less likely to gain a dominant position and fully activate their ovaries [44]. Therefore, the treatments created a set of randomly selected, same-age workers (the focal workers) that, when groups were established, shared the same social experience but were manipulated to diverge in their ovarian activation levels, with F+ focal workers developing higher levels of ovarian activation than F− focal workers (electronic supplementary material, figure S1).

Using 12 *B. terrestris* colonies, we created 80 F+ and 80 F− three-worker groups. In each treatment, 50 groups were used for the measurement of workers' longevity and 30 were used to confirm workers' levels of ovarian activation (see below). All groups were reared in standard conditions identical to those described above (under ‘Colony rearing’).

#### (ii) Measurement of worker longevity and behaviour

To measure worker longevity (in focal and non-focal workers), each group was observed every day for 30 s twice a day (i.e. in the morning then in the afternoon), from the day of group establishment until the last worker's death. The identity of all dead workers, their date of death and all observed occurrences of egg-laying were recorded. Worker longevity was defined as the interval in days between worker eclosion and death. All dead workers were removed and individually stored at −20°C.

To measure potential effects of the treatments on worker behaviour, a subset of 20 groups per treatment was chosen randomly from the 50 groups used to measure worker longevity. These groups were observed for 10 min per group twice a day (morning/afternoon), regularly during the first three months after group establishment (mean ± s.d. interval between observation days = 5.5 ± 2.4 days). During these observations, instantaneous sampling of non-agonistic worker behaviours (including egg-laying) and of workers' spatial location was performed. In addition, throughout the 10-min sampling period, all agonistic behaviours performed and received by the focal workers were recorded. For details of all behaviours measured, and of the categories of spatial location, see the electronic supplementary material.

#### (iii) Ovary dissections and wing cell measurements

Of the 30 groups per treatment set aside for confirmation of workers' level of ovarian activation, 10 were sampled sequentially in three time steps (15, 30 and 60 days after group establishment). All workers (focal and non-focal) were dissected following the methods used in Experiment 1 except that, to assess the level of ovarian activation, the length of all eight terminal oocytes was measured. In the other 50 groups per treatment, workers collected on their death were also dissected. This was to determine the relationship between ovarian activity and longevity in the experimental conditions. The body size of all dissected workers was also estimated from wing cell measurements as described in Experiment 1. All ovarian dissections and wing cell measurements were performed blindly with respect to worker treatment and worker longevity.

### (d) Statistical analyses

Workers in which oocytes could not be accurately measured because of tissue deterioration in ovaries (*n* = 25 of 194 workers and *n* = 39 of 480 workers from Experiment 1 and 2, respectively) were excluded from all analyses involving workers' ovarian activation level. Since workers, including newly eclosed workers, need at least 7 days to fully activate their ovaries [40,41], workers that were under 7 days old when they died were also excluded from these analyses (*n* = 8 workers excluded from Experiment 1).

In both experiments, correlations between worker level of ovarian activation at death and worker longevity were calculated using Spearman's rank correlation tests. Workers' longevity was compared between treatments using Cox's proportional hazards survival analysis. In Experiment 1, workers with activated ovaries were found to be significantly larger than workers with inactivated ovaries (electronic supplementary material). Therefore, the initial model included both workers' ovarian activation level (activated versus inactivated) and body size as predictors. Because the queen can affect levels of ovarian activation in workers [45], models were stratified by the degree of queen presence during the workers' lifetime (constant presence, constant absence or mixed presence/absence), which significantly improved the fit of the data to the models (likelihood ratio test, *p* < 0.0001). Models were clustered at the colony level to account for the presence of correlated observations. In Experiment 2, all models included treatment (F+ versus F−) as a predictor. Preliminary analyses revealed an association, within groups, between longevity of any one worker and the mean longevity of the two other workers (all Spearman's *r* > 0.43, all *p* < 0.0001), suggesting that the presence or absence of group-mates can affect workers' longevity. Number of group-mates (0, 1 or 2) was thus included as a time-dependent covariate (updated daily) [46], which significantly improved the fit of the data to the models (likelihood ratio tests, *p* < 0.001). Separate models were run for focal and non-focal workers and the model for non-focal workers was clustered at the group level to account for the presence of correlated observations (two non-focal workers were present within each group). Non-focal workers, but not focal workers, were found to differ significantly in body size across treatments (electronic supplementary material). Body size was therefore included in the non-focal worker model. However, because Cox model assumption tests indicated large deviations from proportional hazards for this factor, body size could not be directly tested as a covariate and so was included as a strata variable in the model (workers were classified as small, medium or large by dividing the distribution of body size into three classes by tercentiles).

In Experiment 2, levels of ovarian activation and relative rates of non-agonistic worker behaviours were analysed with linear models (LMs) or linear mixed model (LMMs) with group membership as a random factor conditional on the presence of dependent observations. Body size was included as a covariate in all models testing differences in levels of ovarian activation. For non-agonistic behaviours, rare activities (representing less than 1% of total acts) were excluded from analysis. Agonistic behaviours provided count data and were thus analysed by generalized linear models (GLMs) using a quasiPoisson error distribution. Egg-laying activity was compared between treatments using *χ*^2^-tests with expected frequencies being weighted by the relative proportion of scans performed on workers of each treatment during the interval between the first and last observed instances of egg-laying.

All analyses were performed with the statistical software R v.3.2.1 using the survival and lme4 packages. Post hoc tests were corrected for multiple comparisons with the sequential Bonferroni–Holm correction procedure and adjusted *p*-values are denoted *p′* (and should be compared with the standard 0.05 significance threshold). Box–Cox transformation was applied to achieve normality and homoscedasticity when necessary. Full details of statistical model fitting and testing of model assumptions are provided in the electronic supplementary material, tables S1–S6.

## 3. Results

### (a) Experiment 1: association of worker longevity and ovarian activation in whole colonies

**Figure 1.**
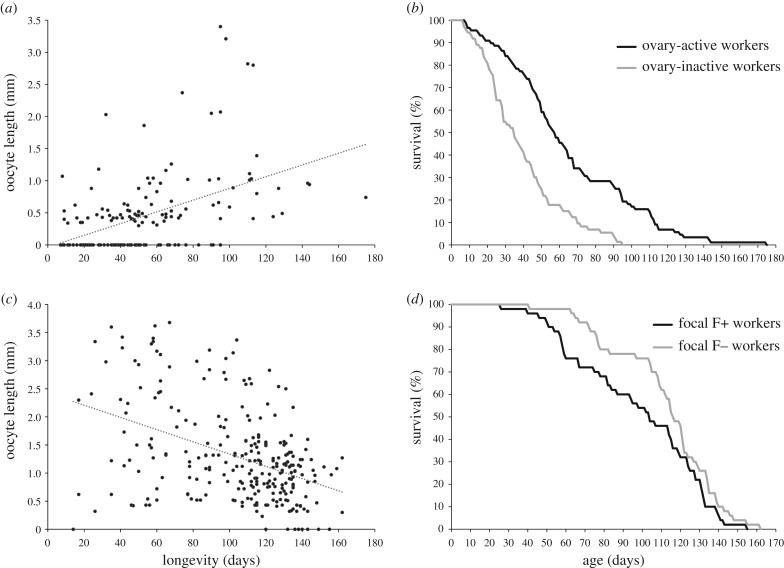
Relationship between level of ovarian activation and longevity or survival in *B. terrestris* workers. (*a*) Relationship between length of longest oocyte (millimetres) and worker longevity (days between worker eclosion and death) in workers in whole colonies (Experiment 1; *n* = 161 workers). Dotted line is linear regression line (for illustrative purposes only). (*b*) Per cent of workers surviving as a function of age (days from eclosion) for ovary-active workers (black line) and ovary-inactive workers (grey line) in whole colonies (Experiment 1; *n* = 88 and 73 workers, respectively). (*c*) Relationship between length of longest oocyte (millimetres) and worker longevity (days between worker eclosion and death) in randomly selected workers (Experiment 2; *n* = 262 workers). Dotted line is linear regression line (for illustrative purposes only). (*d*) Per cent of workers surviving as a function of age (days from eclosion) for focal F+ workers (black line), i.e. workers manipulated to be more ovary-active, and focal F− workers (grey line), i.e. workers manipulated to be less ovary-active (Experiment 2; *n* = 50 and 50 workers, respectively). For statistical analyses, see ‘Results’.

There was a significant positive relationship between workers' level of ovarian activation and longevity in all three classes of workers (*r* = 0.37, *p* < 0.001, *n* = 82; *r* = 0.44, *p* = 0.024, *n* = 26; *r* = 0.49, *p* < 0.001, *n* = 53; in workers that experienced the queen's constant presence, constant absence or mixed presence/absence, respectively). Correspondingly, there was also a significant positive relationship between workers' level of ovarian activation and longevity across all classes of worker (figure 1*a*; *r* = 0.49, *p* < 0.0001, *n* = 161). This relationship was also significantly positive for ovary-active workers alone, i.e. workers that had at least one identifiable oocyte in their ovaries (*r* = 0.43, *p* < 0.0001, *n* = 88).

The Cox survival analysis demonstrated a significant effect of ovarian activation (electronic supplementary material, table S1; *χ*^2^ = 35.6, d.f. = 1, *p* < 0.0001, *n* = 161) but no significant effect of body size (electronic supplementary material, table S1; *χ*^2^ = 0.05, d.f. = 1, *p* = 0.82, *n* = 153) on workers' survival. Workers with activated ovaries had higher survival than workers with non-activated ovaries (figure 1*b*; hazard ratio (95% confidence interval) = 0.35 (0.25–0.50)). Hence, overall, Experiment 1 showed that workers' ovarian activation in whole colonies was associated with increased worker longevity.

### (b) Experiment 2: association of worker longevity and ovarian activation in randomly selected workers

#### (i) Ovarian activation and egg-laying

Among focal workers from the 60 groups providing worker samples for dissection, F+ workers exhibited a significantly higher level of ovarian activation than F− workers consistently across all three time steps (electronic supplementary material, figure S2 and table S2; LM: *χ*^2^ = 10.20, d.f. = 1, *p* = 0.0014, *n* = 60). By contrast, among the non-focal workers from these groups, F+ and F− workers did not differ significantly in their levels of ovarian activation (electronic supplementary material, figure S3 and table S3; LMM: *χ*^2^ = 1.42, d.f. = 1, *p* = 0.23, *n* = 119). Overall, F+ focal workers exhibited a significantly higher level of ovarian activation than all other worker groups (electronic supplementary material, figure S4 and table S4; LMM: *χ*^2^ = 16.18, d.f. = 3, *p* = 0.001, *n* = 179; post hoc tests, all *p*' < 0.021).

During scans of the experimental groups, a total of 78 instances of egg-laying were recorded. Among focal workers, F+ workers performed egg-laying significantly more often than F− workers (totals of 22 versus 12 egg-laying acts, respectively; *χ*^2^ = 4.03, d.f. = 1, *p* = 0.045). By contrast, among non-focal workers, F+ and F− workers did not differ significantly in the number of egg-laying acts performed (totals of 15 versus 29 acts, respectively; *χ*^2^ = 3.54, d.f. = 1, *p* = 0.06). Overall, these results showed that, as planned, the social treatment led to F+ focal workers having higher levels of ovarian activation and egg-laying than F− focal workers.

#### (ii) Worker longevity

In contrast with the results of Experiment 1, there was a significant negative relationship between workers' level of ovarian activation and longevity in all four groups of workers (*r* = −0.65, *p* < 0.001, *n* = 41; *r* = −0.41, *p* = 0.005, *n* = 45; *r* = −0.32, *p* = 0.002, *n* = 89; *r* = −0.27, *p* = 0.013, *n* = 87; in focal F+, focal F−, non-focal F+ and non-focal F− workers, respectively). Hence the worker group showing the strongest negative relationship between workers' level of ovarian activation and longevity (F+ focal workers) was the group with the highest level of ovarian activation (see above). Given that all four worker groups showed the same relationship, there was also a significant negative relationship between workers' level of ovarian activation and longevity across all workers combined (figure 1*c*; *r* = −0.39, *p* < 0.0001, *n* = 262).

In focal workers, the Cox survival analysis demonstrated a significant effect of treatment (electronic supplementary material, table S5; *χ*^2^ = 3.86, d.f. = 1, *p* = 0.049, *n* = 100) on workers' survival. Workers in the F+ treatment had lower survival than workers in the F− treatment (figure 1*d*; hazard ratio (95% confidence interval) = 1.47 (1.01–2.15)). In non-focal workers, there was no significant effect of treatment on workers' longevity (electronic supplementary material, table S6; *χ*^2^ = 0.47, d.f. = 1, *p* = 0.49, *n* = 200). Hence, the combined ovary dissection and survival data from Experiment 2 showed that, in contrast to the results of Experiment 1, workers' ovarian activation in randomly selected workers was associated with reduced longevity (figure 1).

Furthermore, the Cox survival analysis showed a significant effect of group size on workers' survival in both focal (electronic supplementary material, table S5; *χ*^2^ = 11.1, d.f. = 1, *p* < 0.001, *n* = 100) and non-focal workers (electronic supplementary material, table S6; *χ*^2^ = 78.4, d.f. = 1, *p* < 0.0001, *n* = 200). In each set of workers, risks of death were reduced with increased group size (hazard ratios (95% confidence interval) = 0.59 (0.43–0.80) and 0.42 (0.35–0.51), respectively).

#### (iii) Behaviour

There were no significant differences between focal F+ and F− workers in either the relative rates of non-agonistic behaviours performed or spatial location (electronic supplementary material, table S7; LM: behaviours: all *p* > 0.37; spatial location: all *p* > 0.057). By contrast, non-focal F+ and F− workers exhibited different relative rates of non-agonistic behaviours (electronic supplementary material, table S8). Non-focal F+ workers remained significantly more inactive (LMM: *χ*^2^ = 4.11, d.f. = 1, *p* = 0.043) and performed significantly less brood care (LMM: *χ*^2^ = 17.60, d.f. = 1, *p* < 0.001) than non-focal F− workers.

A total of 180 agonistic acts were recorded, with alarm, threatening and overt aggression representing 46.1%, 40.6% and 13.3% of such acts, respectively. Most agonistic acts (134/180) were performed during the first two weeks after group establishment. Analyses showed that F+ and F− focal workers exhibited no significant differences in the numbers of alarm and overt aggressive behaviours performed and received (electronic supplementary material, table S9; GLM: all *p* > 0.20). However, consistent with their greater level of ovarian activation, F+ focal workers performed significantly more, and received significantly less, threatening behaviours than F− focal workers (electronic supplementary material, table S9; GLM: both *p* < 0.001).

## 4. Discussion

### (a) Costs of reproduction and worker quality in *Bombus terrestris*

We investigated how reproduction affects longevity among workers of the annual eusocial bumblebee *B. terrestris*. We found that, in whole colonies, workers' level of ovarian activation was significantly positively associated with longevity and that ovary-active workers lived significantly longer than ovary-inactive workers (Experiment 1; figure 1). However, when workers were experimentally forced to reproduce, workers' level of ovarian activation was significantly negatively associated with longevity and ovary-active workers had significantly reduced longevities relative to those of ovary-inactive workers (Experiment 2; figure 1).

In Experiment 2, workers were randomly allocated to either reproductive or non-reproductive treatments, thereby decoupling reproduction from other traits. The reduced longevity of reproductive workers in this experiment therefore shows that reproduction incurs survival costs in *B. terrestris* workers. These costs appear to be intrinsic to individuals or groups, because all individuals in both treatments were kept in complete confinement under the same conditions. In Experiment 1, by contrast with Experiment 2, workers could freely ‘choose’ whether to become reproductive or not. This suggests that workers which become reproductive in whole colonies are capable of overcoming the costs of reproduction, so generating the positive association between level of ovarian activation and longevity observed. In turn, this suggests that these workers are intrinsically high-quality individuals, consistent with the known link between dominance and reproduction in workers of eusocial Hymenoptera, including *B. terrestris* [29]. Overall, therefore, our findings show that reproductive workers in *B. terrestris* experience costs of reproduction as in non-social organisms and that therefore they do not exhibit a genuine reversal of the negative fecundity–longevity trade-off. In insects in general, a genetic and endocrine network involving insulin-like/IGF-1 signalling, juvenile hormone, vitellogenin and/or other yolk proteins is thought to mediate the fecundity–longevity trade-off [28]. Hence a corollary of our findings is that, at the proximate (mechanistic) level, *B. terrestris* workers by default exhibit conventional relationships among the elements of this network, and that any remodelling of the network (leading to a reversed fecundity–longevity trade-off) occurs in high-quality individuals only. At the ultimate (evolutionary) level, reproductive workers might benefit from living longer because, as in queens, it would facilitate their achieving higher offspring production, i.e. greater direct fitness.

These findings raise the question of what intrinsic quality might entail in *B. terrestris* workers. That intrinsic quality varies among workers and that high quality is linked with ovarian activation are points consistent with previous observations that only a proportion (28–45%) of all *B. terrestris* workers lay eggs in whole colonies [40]. They are also consistent with our finding that longer-lived, ovary-active workers were significantly larger than shorter-lived, ovary-inactive ones in Experiment 1 (electronic supplementary material), matching associations of body size and quality in other eusocial Hymenoptera [47]. In *B. hypnorum*, positive associations were reported between workers' reproductivity, body size and fat body size [48]. Since the fat body is the site of vitellogenin biosynthesis [49], and vitellogenin is overexpressed in the fat body of ovary-active *B. terrestris* workers [26], we hypothesize that, in the present context, intrinsic quality in workers is a function of fat body size and level of biosynthetic activity. Recent findings suggesting an uncoupling of juvenile hormone and vitellogenin in reproductive *B. terrestris* workers [44] might then reflect the remodelling of conserved genetic and endocrine networks hypothesized to mediate effects on ageing and longevity in such workers.

### (b) Additional factors potentially affecting differential worker longevity

At the proximate level, two potential additional explanations for the reduced longevity of ovary-active workers observed in Experiment 2 can be ruled out. First, the reduced longevity of ovary-active workers potentially arose from differences in relative rates of non-agonistic behaviours performed. However, F+ and F− focal workers performed all these behaviours at similar rates. Therefore, unlike the case in the ant *Diacamma* sp., in which greater workload may account for the reduced longevity of non-reproductive workers [32], the longevity difference between reproductive and non-reproductive workers did not result from differences in workload. Moreover, as F+ focal workers did not seek more food than focal F− workers, reproductive individuals did not appear to attempt to compensate for individual costs of reproduction by obtaining a greater share of group-level benefits, as has been suggested for ant queens [23].

Second, the reduced longevity of ovary-active workers in Experiment 2 potentially arose from differences in rates of agonistic behaviours. This is also unlikely, as (i) focal F− workers received more agonistic behaviour (significantly more in the case of threatening behaviour) and yet had greater longevity and (ii) overt aggression was relatively rare and most agonistic acts were performed during the first two weeks after group establishment, yet median longevity of all workers was much greater than this (112 days; electronic supplementary material). In addition, differences in rates of agonistic behaviours are also unlikely to explain longevity differences because, in Experiment 1, ovary-active workers had the greatest longevity, and overtly aggressive behaviours are expressed almost entirely by and towards reproductive workers in whole colonies of *B. terrestris* [50].

### (c) Effect of group size

Our findings showed that workers in Experiment 1 were shorter-lived on average than workers in Experiment 2 (electronic supplementary material). The median longevity of workers in Experiment 1 (46 days) was close to those previously measured in whole *B. terrestris* colonies (*ca* 25–45 days: [51]), whereas the median longevity of workers in Experiment 2 was 112 days (electronic supplementary material). Given that workers in the two experiments were kept in whole colonies and three-worker groups, respectively, these findings are consistent with a previous study that documented the occurrence of greater worker longevity in smaller groups in the honeybee *Apis mellifera* [52]. Interestingly, however, we found the opposite pattern within the small worker groups of Experiment 2, in which greater longevity was associated with larger groups. Hence, group size seems to affect longevity in bumblebee workers non-linearly. So far, nothing is known about the mechanism by which group size might affect worker longevity in eusocial insects. Therefore, along with those of Rueppell *et al*. [52], our findings suggest that the effects of group size on individual longevity in eusocial insect colonies would repay further study.

## 5. Conclusion and implications

We conclude that *B. terrestris* workers experience costs of reproduction and accordingly we hypothesize that intrinsic quality differences between individuals account for the apparently reversed fecundity–longevity trade-off among workers in whole colonies. In addition, only high-quality workers overcoming the costs of reproduction may exhibit a remodelling of conserved genetic and endocrine networks underpinning reproduction and longevity, suggesting that such remodelling may be condition-dependent even within a single caste phenotype.

*Bombus terrestris* queens show a positive association of longevity and sexual production [22], suggesting that, as a class, they exhibit a positive association of longevity and fecundity, i.e. a reversed fecundity–longevity trade-off as suggested in queens of other eusocial species (see ‘Introduction’). However, our findings raise the question of whether the fecundity–longevity trade-off is truly reversed in *B. terrestris* queens, and by extension in social insect queens in general, or whether an effect of quality applies in queens as in workers. Three points are relevant here. First, it is already well recognized that, in non-social organisms, deviations from a negative fecundity–longevity association can occur when individuals vary in intrinsic quality and/or resources held [4,5]. Because high-quality (well-resourced) individuals then both reproduce more and live longer than poor-quality ones, between-individual comparisons yield a positive association of fecundity and longevity, even though costs of reproduction are not abolished and investment between reproduction and maintenance is still traded-off within individuals [4,5]. Second, queen-worker caste determination in eusocial insects almost always entails queens receiving greater levels of nutrition as larvae, and also sometimes qualitatively different nutrients [53]. Combined with our current results, these points suggest the hypothesis that both queens and workers in eusocial insects represent a special case of intrinsic quality differences (condition-dependence) in individuals generating a positive fecundity–longevity association, as found in non-social organisms when quality and/or resources vary. Third, the finding of Schrempf *et al*. [24] that *Cardiocondyla* ant queens lack costs of reproduction implies that there is indeed a true reversal of the fecundity–longevity trade-off in queens in at least some social insects. In this respect, it is conceivable that the fecundity–longevity association may evolve differently according to whether the colony cycle is perennial (as in ants) or annual (as in *Bombus*). However, given that reproductive *B. terrestris* workers appear to exhibit costs of reproduction, there is evidently a need to test for such costs experimentally in queens of other species [38], including *B. terrestris*.

If eusocial insect queens, in any species, are a special case of condition-dependence affecting the fecundity–longevity association, this would imply that the view that queens exhibit a truly reversed trade-off needs to be re-examined. If not, then one would conclude that, at least in *B. terrestris*, reproduction in the queen and worker castes affects ageing and longevity differentially. Both these possibilities would have major implications for our understanding of how social evolution interacts with the evolution of ageing. The first would imply that eusociality has not necessarily entailed a fundamental remodelling of conserved genetic and endocrine networks regulating ageing and associated traits. The second would imply that selection can lead to fecundity–longevity associations reflecting differential remodelling of genetic and endocrine networks across different caste phenotypes arising from a single genome. Therefore, as advocated by Korb [38], extending experimental approaches to these issues promises to yield further informative results.

## Supplementary Material

Supplementary methods and results

## Data accessibility

Data are available in Dryad at: http://dx.doi.org/10.5061/dryad.9jh21 [54].
