# Supplementary material for: Evolution of ageing, costs of reproduction and the fecundity–longevity trade-off in eusocial insects
Source: Proc Biol Sci. 2017 Jul 12;284(1858):20170380. doi: 10.1098/rspb.2017.0380 (PMC5524490; doi:10.1098/rspb.2017.0380)
Supplement: Supplementary methods and results [file rspb20170380supp1.pdf]

Evolution of ageing, costs of reproduction and the fecundity-longevity trade-off in eusocial insects

Pierre Blacher[1,2], Timothy J. Huggins[1], Andrew F. G. Bourke[1]

[1]School of Biological Sciences, University of East Anglia, Norwich Research Park, Norwich NR4 7TJ, UK
[2]Present address: Department of Ecology and Evolution, University of Lausanne, Bâtiment Biophore, Quartier UNIL-Sorge, 1015 Lausanne, Switzerland

Proceedings of the Royal Society B 10.1098/rspb.2017.0380

Electronic Supplementary Material

1. Supplementary Methods

All colonies were of *Bombus terrestris audax* and were obtained from Biobest® Biological Systems, Belgium NV. They were transferred to wooden nest-boxes with internal dimensions, 17 cm × 27.5 cm × 16 cm high, and with a clear Perspex lid. Dried pollen and artificial nectar ('Attracker', Koppert Biological Systems, Haverhill, UK) were provided *ad libitum*.

(a) Experiment 1: association of worker longevity and ovarian activation in whole colonies

Colony set-up and measurement of worker longevity
Twenty-five colonies of *B. t. audax* were obtained from the supplier on 27 February 2013. The experiment ended on 28 August 2013, when only 1–2 living workers remained in any colony.

Initial worker marking was conducted on 1 March 2013 and marking of newly-eclosed workers was continued until 14 May 2013 (or until an earlier date if no more workers were produced) and for most colonies was conducted over two periods, with an interval of 8–38 days between them in which no newly-eclosed workers were marked. Two periods were used for marking so that workers eclosing and dying at different phases in the colony cycle could be included in the estimates of worker longevity.

During the inspections of each colony, any adult sexuals (new queens and males) were removed (along with any marked, dead workers). This was because, in nature, adult sexuals leave the natal nest after a few days [1].

The queen removals (in selected colonies) were conducted on 17 April 2013. In two colonies (colonies 10 and 17), the colony queen died naturally before queen removals had been conducted. Therefore, to obtain a sample of ten queenless colonies, we removed the colony queen from a random sample of eight of the remaining 23 queenright colonies (i.e. those with a colony queen).

Ovary dissections and wing cell measurements
Ovary dissections were performed in Ringer solution under a Zeiss Discovery V12 Stereomicroscope linked to a monitor. The length of the single longest visible oocyte (usually but not always the terminal oocyte) within the two ovaries (or only one if the second ovary was not visible) was measured using the measuring tool in the Zeiss AxioVision 4 software package. For all dissected workers, the same method was used to measure the length of the radial cell of the right forewing as an index of total body size, or the length of the radial cell of the left forewing if the right forewing was missing or damaged [2]. All ovarian dissections and wing cell measurements were performed blindly with respect to worker longevity.

Ovary dissections and wing cell measurements were carried out on a total of 392 workers across 20 colonies, these workers having been selected on the basis of each (a) having known eclosion and death dates and hence a known longevity, (b) falling representatively into colonies in which the queen was present and alive or colonies in which the queen was removed or had died, and (c) falling representatively into cohorts eclosing or dying before or after the date of the queen's removal or death (see main text, 'Sample details' for further details). Of these workers, we then selected all workers with at least one value for oocyte length or wing cell length (n = 194). This sample was less than the initial sample because in

some workers (*n* = 223) oocyte length could not be measured and ovarian status (active v. inactive) could not be scored because of tissue deterioration. Such deterioration almost certainly arose because workers could not be collected for dissection immediately after death in all cases. In addition, in some workers (*n* = 204), wing cell measures were not taken either because both wings were missing or damaged or because the ovaries were not scorable. Workers in the selected sample included individuals falling within the entire range of worker longevities found in the initial sample (selected sample: range = 2 to 175 days, *n* = 194 workers; initial sample: range = 2 to 175 days, *n* = 392 workers).

(b) Experiment 2: association of worker longevity and ovarian activation in randomly selected workers

Experimental treatments
Fourteen colonies of *B. t. audax* were obtained from the supplier on 20 November 2014. The experiment ended on 21 May 2015, when the last living experimental worker died.

Colonies were monitored daily to track their developmental stage and specifically to detect whether they had reached the competition point (start of worker egg-laying). The competition point was defined as occurring when at least one of the following events was observed: two egg-cells or more opened for at least two consecutive days, worker egg-laying or worker egg-eating [3]. All workers used to establish the experimental groups of three workers were taken from pre-competition point colonies. Two colonies failed to produce sufficient numbers of newly-eclosed workers when the experimental groups were being established. Therefore, workers were drawn from up to 12 of the 14 colonies. In the 100 groups used for measuring workers' longevity, the median (range) number of workers used per colony as focal workers was 4 (2-10) from 11 colonies for F+ workers and 4 (1-9) from 12 colonies for F- workers.

The plastic boxes used to house the experimental groups of three workers were each 7.9 cm × 13.8 cm × 5.1 cm high and, as well as the workers, contained a single cocoon (in which was a living or frozen larva or pupa) to stimulate egg-laying (of focal workers in the F+ treatments and of non-focal workers in the F- treatment). During the experiment, any adult males eclosing from brood produced by the workers were removed.

Measurement of worker longevity and behaviour
Instantaneous sampling of behaviour and spatial location was conducted every 2 minutes during each 10-minute sampling period. Non-agonistic worker behaviours recorded were: patrolling the nest-box, inactivity (immobility over several seconds), collecting food (pollen or artificial nectar), brood care (feeding larvae, manipulating the wax of larval cells), building nest structures (manipulating wax on honey pots and egg-cells, producing covering wax), wing fanning and egg laying. The spatial location of each worker was recorded as worker presence on either the brood, on the feeding cups or in an empty zone.

Agonistic behaviours performed and received by the focal workers were recorded throughout each 10-minute sampling period and were classified into three categories according to their level of aggressiveness: (i) alarm behaviours, including buzzing (fast, brief wing vibrations while opposite another worker) and humming (a series of wing vibrations lasting less than 3 s, performed by workers while they are active); (ii) threatening behaviours, including darting movements and movement of the front legs while facing another worker; and (iii) overtly aggressive behaviours, including head butting, biting and stinging attempts directed at another worker.

For analyses of relative rates of non-agonistic worker behaviours, only data from workers observed on at least 9 different days (= 90 scans) were used (mean numbers of scans per worker ± SE were: focal workers, 146.5 ± 4.2, n = 20, and 151.7 ± 2.3, n = 20, for F+ and F- social treatments, respectively; non-focal workers, 138.3 ± 3.9, n = 38, and 144.4 ± 2.9, n = 36, for F+ and F- social treatments, respectively).

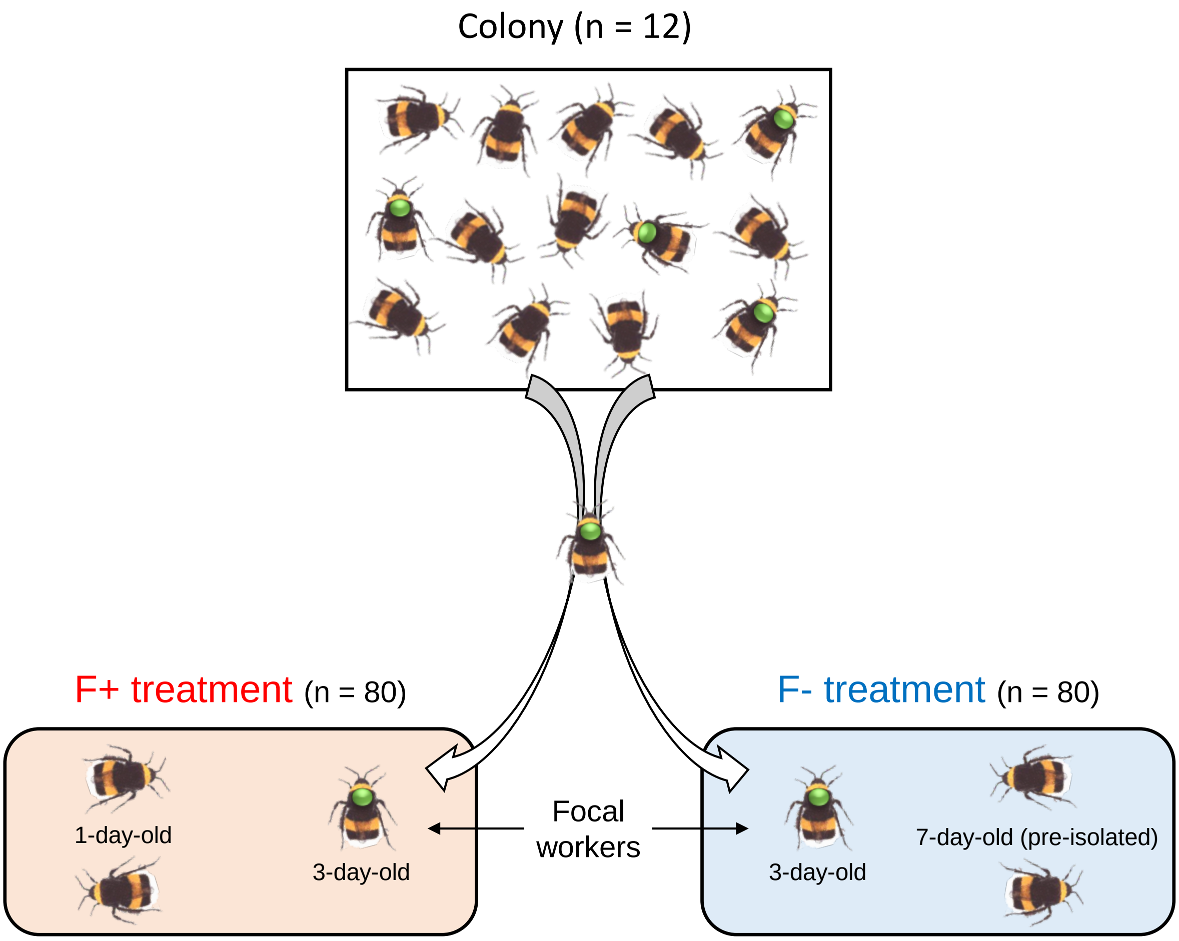

Figure S1. Experimental treatments in Experiment 2. Marked 3-day-old focal workers were randomly assigned to either the F+ treatment (i.e. transferred to a box with two 1-day-old sister-workers) or the F- treatment (i.e. transferred to a box with two 7-day-old sister-workers that had themselves previously been isolated as a pair for the previous 5 days). See main text for further details.

2. Supplementary Results

(a) Experiment 1: association of worker longevity and ovarian activation in whole colonies

Minimum and maximum longevities among all workers (n = 194) were 2 and 175 days, respectively, with a mean ± SD of 49.9 ± 32.9 days and a median of 46 days (quartiles 25, 67.75 days). Minimum and maximum longevities among ovary-active workers (n = 88) were 8 and 175 days, respectively, with a mean ± SD of 64.7 ± 35.1 days and a median of 57.5 days (quartiles 41.75, 91.75 days). Minimum and maximum longevities among ovary-inactive workers (n = 73) were 7 and 95 days, respectively, with a mean ± SD of 38.9 ± 22.1 days and a median of 35 days (quartiles 24, 50 days).

Ovary-active and ovary-inactive workers differed significantly in body size (ANOVA: $F_{1,151}$ = 5.92, p = 0.016), with ovary-active workers being larger than ovary-inactive workers (mean length of the radial cell ± SE: 2.49 ± 0.03 mm, n = 84 and 2.36 ± 0.04 mm, n = 69, respectively).

Table S1. Successive Cox proportional hazards models fitted to test the effects of ovarian activation level at death (active/inactive ovaries) on workers' longevity in Experiment 1 (see figure 1b). Results of proportional hazards assumption tests based on the scaled Schoenfeld residuals are given in the right-hand side of the table (significant results indicate deviation from proportional hazards). Models 2 and 3 include degree of queen presence during the workers' lifetime (constant presence, constant absence or mixed presence/absence) as a strata variable. Last model is the one retained for analysis. * HR = Hazard ratio (95 % lower and upper confidence intervals).

Models	Factors	HR (95% CI)*	χ^2	df	*p*	rho	χ^2	*p*
Model 1	Ovarian activation (0 = inactive, 1 = active)	0.41 (0.25-0.66)	13.6	1	0.0002	-0.09	3.46	0.063
	Body size	0.50 (0.22-1.14)	2.7	1	0.1	0.126	8.11	0.004
	GLOBAL		53.69	2	<0.0001	NA	8.21	0.016
Model 2	Ovarian activation (0 = inactive, 1 = active)	0.35 (0.25-0.51)	31.8	1	<0.0001	-0.01	0.016	0.90
	Body size	0.92 (0.44-1.93)	0.05	1	0.82	0.085	2.21	0.14
	GLOBAL		37.14	2	<0.0001	NA	2.38	0.30
Model 3	Ovarian activation (0 = inactive, 1 = active)	0.35 (0.25-0.50)	35.58	1	<0.0001	0.036	0.191	0.66

(b) Experiment 2: association of worker longevity and ovarian activation in randomly selected workers

Minimum and maximum longevities among all workers (*n* = 300) were 14 and 162 days, respectively, with a mean ± SD of 102.7 ± 33.0 days and a median of 112 days (quartiles 78, 129 days). Comparing across all workers in both experiments, workers in Experiment 2 therefore had significantly greater longevity than those in Experiment 1 (ANOVA: $F_{1, 492} = 307.7$, $p < 0.0001$).

Among focal workers, mean longevities ± SD of F+ (*n* = 50) and F- (*n* = 50) workers were 97.9 ± 33.4 and 112.5 ± 26.8 days, respectively, and medians were 104 days (quartiles 67, 127.25) and 116.5 days (quartiles 104.25, 133), respectively. Among non-focal workers, mean longevities ± SD of F+ (*n* = 100) and F- (*n* = 100) workers were 94.9 ± 35.5 and 107.9 ± 31.2 days, respectively, and medians were 103.5 days (quartiles 59.75, 124.25) and 116 days (quartiles 89, 131), respectively. There was no significant effect of colony of origin on focal workers' longevity (ANOVA: $F_{11,88} = 1.69$, $p = 0.089$, $n = 100$).

Among focal workers, F+ and F- workers did not differ significantly in body size (ANOVA: $F_{1, 98} = 3.1$, $p = 0.07$; mean length of the radial cell ± SE: 2.82 ± 0.03 mm, *n* = 50 and 2.89 ± 0.03 mm, *n* = 50, respectively). By contrast, F+ and F- non-focal workers differed significantly in body size (ANOVA: $F_{1,198} = 13.1$, $p < 0.001$), with non-focal F+ workers being larger than non-focal F- workers (mean length of the radial cell ± SE: 2.89 ± 0.02 mm, *n* = 100 and 2.79 ± 0.02 mm, *n* = 100, respectively).

Table S2. Successive linear models fitted to test the effects of treatment (F+/F-) on ovarian activation levels of focal workers from the groups set aside for confirmation of workers' level of ovarian activation in Experiment 2 (see figure S2). Last model is the one retained for analysis.

Models	Factors	χ^2	df	*p*
Model 1	Treatment	9.75	1	0.0018
	Body size	4.61	1	0.032
	Time	0.54	2	0.76
	Treatment x Time	1.55	2	0.46
Model 2	Treatment	9.83	1	0.0017
	Body size	4.31	1	0.038
	Time	0.55	2	0.76
Model 3	Treatment	10.2	1	0.0014
	Body size	5.41	1	0.02

Table S3. Successive linear mixed models fitted to test the effects of treatment (F+/F-) on ovarian activation levels of non-focal workers from the groups set aside for confirmation of workers' level of ovarian activation in Experiment 2 (see figure S3). Last model is the one retained for analysis.

Models	Factors	χ^2	df	*p*
Model 1	Treatment	1.78	1	0.18
	Body size	5.35	1	0.02
	Time	4.78	2	0.09
	Treatment x Time	4.29	2	0.12
Model 2	Treatment	1.74	1	0.187
	Body size	6.44	1	0.011
	Time	4.69	2	0.096
Model 3	Treatment	1.42	1	0.23
	Body size	4.69	1	0.03

Table S4. Successive linear mixed models fitted to test the effects of worker condition on ovarian activation levels of all workers from the groups set aside for confirmation of workers' level of ovarian activation in Experiment 2 (see figure S4). Worker condition refers to the four different worker types (Focal F+, Focal F-, Non-focal F+ and Non-focal F-). Last model is the one retained for analysis.

Models	Factors	χ^2	df	*p*
Model 1	Worker condition	16.19	3	0.001
	Body size	10.9	1	0.001
	Time	1.83	2	0.4
	Worker condition x Time	5.21	3	0.16
Model 2	Worker condition	16.42	3	0.0009
	Body size	10.56	1	0.0012
	Time	1.77	2	0.41
Model 3	Worker condition	16.18	3	0.001
	Body size	9.37	1	0.002

Table S5. Successive Cox proportional hazards models fitted to test the effects of treatment (F+/F-) on focal workers' longevity in Experiment 2 (see figure 1d). Results of proportional hazards assumption tests based on the scaled Schoenfeld residuals are given in the right-hand side of the table (significant results indicate deviation from proportional hazards). Last model is the one retained for analysis. * HR = Hazard ratio (95 % lower and upper confidence intervals).

Models	Factors	HR (95% CI)*	χ^2	df	*p*	rho	χ^2	*p*
Model 1	Treatment (0 = F-, 1 = F+)	1.43 (0.97-2.11)	3.21	1	0.073	-0.114	1.22	0.27
Model 2	Treatment (0 = F-, 1 = F+)	1.47 (1.01-2.15)	3.86	1	0.049	-0.055	0.277	0.6
	Group size	0.59 (0.43-0.80)	11.1	1	0.0008	-0.076	0.666	0.41
	GLOBAL		15.68	2	0.0004	NA	0.903	0.64

Table S6. Successive Cox proportional hazards models fitted to test the effects of treatment (F+/F-) on non-focal workers' longevity in Experiment 2. Results of proportional hazards assumption tests based on the scaled Schoenfeld residuals are given in the right-hand side of the table (significant results indicate deviation from proportional hazards). The factor 'body size' did not meet the assumption of proportional hazards (see model 2) and was therefore included as a strata variable in the following model (model 3). Last model is the one retained for analysis. * HR = Hazard ratio (95 % lower and upper confidence intervals).

Models	Factors	HR (95% CI)*	χ^2	df	*p*	rho	χ^2	*p*
Model 1	Treatment (0 = F-, 1 = F+)	1.25 (0.89-1.75)	1.7	1	0.19	-0.144	5.74	0.017
	Body size	1.12 (0.52-2.43)	0.09	1	0.77	-0.169	8.33	0.004
	GLOBAL		1.96	2	0.38	NA	16.77	0.0002
Model 2	Treatment (0 = F-, 1 = F+)	1.08 (0.81-1.45)	0.3	1	0.58	-0.107	2.32	0.13
	Body size	0.71 (0.34-1.45)	0.91	1	0.34	-0.242	15.61	<0.0001
	Group size	0.44 (0.36-0.53)	73.00	1	<0.0001	0.081	1.29	0.25
	GLOBAL		87.79	3	<0.0001	NA	22.54	<0.0001
Model 3	Treatment (0 = F-, 1 = F+)	1.11 (0.83-1.48)	0.47	1	0.49	-0.045	0.396	0.53
	Group size	0.42 (0.35-0.51)	78.4	1	<0.0001	0.032	0.195	0.66
	GLOBAL		94.02	2	<0.0001	NA	0.884	0.64

Table S7. Relative rates of non-agonistic worker behaviours and (final two rows) spatial location of focal workers according to treatment (Experiment 2). Results are presented as mean ± SE percentage of scans per worker in which a given behaviour was performed or a given spatial location occupied. All d.f. = 1.

	F+ workers (*n* = 20)	F- workers (*n* = 20)	χ^2	*p*
Patrolling the nest-box	35.49 ± 1.42	34.55 ± 1.43	0.22	0.64
Inactivity	39.25 ± 1.95	41.92 ± 2.19	0.80	0.37
Collecting food	5.74 ± 0.89	5.84 ± 0.84	0.02	0.87
Brood care	18.35 ± 1.92	16.44 ± 1.58	0.48	0.49
Presence on the brood	82.10 ± 2.48	76.67 ± 2.32	3.63	0.057
Presence on the feeding cups	5.98 ± 0.72	7.51 ± 0.69	2.55	0.11

Table S8. Relative rates of non-agonistic worker behaviours of non-focal workers according to treatment (Experiment 2). Results are presented as mean ± SE percentage of scans per worker in which a given behaviour was performed. All d.f. = 1.

	F+ workers (*n* = 38)	F- workers (*n* = 36)	χ^2	*p*
Patrolling the nest-box	36.14 ± 1.44	34.07 ± 1.23	0.88	0.35
Inactivity	45.26 ± 1.59	40.36 ± 1.14	4.11	0.043
Collecting food	5.08 ± 0.41	5.84 ± 0.45	1.48	0.22
Brood care	12.27 ± 0.93	18.17 ± 1.03	17.60	< 0.001

Table S9. Agonistic behaviours performed and received by focal workers (Experiment 2). Results are presented as mean ± SE occurrences of behaviours per worker. All d.f. = 1.

Behaviours		F+ workers (*n* = 20)	F- workers (*n* = 20)	χ^2	*p*
Alarm	Performed	0.70 ± 0.27	0.65 ± 0.32	0.01	0.91
	Received	1.25 ± 0.67	1.55 ± 0.37	0.14	0.70
Threatening	Performed	1.85 ± 0.47	0.25 ± 0.10	17.57	< 0.001
	Received	0.20 ± 0.12	1.35 ± 0.26	15.92	< 0.001
Overt aggression	Performed	0.50 ± 0.22	0.20 ± 0.12	1.58	0.21
	Received	0.15 ± 0.11	0.35 ± 0.15	1.14	0.29

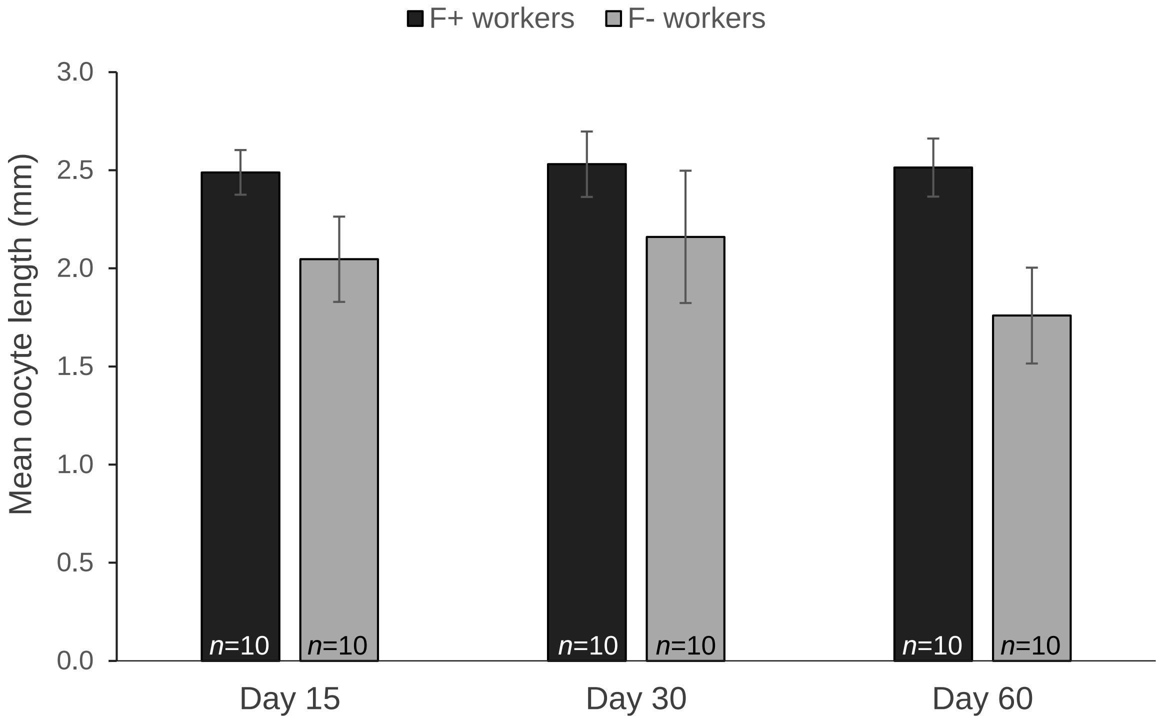

Figure S2. Levels of ovarian activation in focal workers from the groups set aside for confirmation of workers' level of ovarian activation (Experiment 2). F+ (black bars) and F- (grey bars) workers were collected at three different time steps (day 15, day 30 and day 60 after group establishment). Level of ovarian activation was measured as the mean length of the eight terminal oocytes. Error bars are ± 1 SE and sample sizes (worker numbers) are inset within the base of each bar. There was no significant interaction between treatment and time (LM: $\chi^2 = 1.55$, d.f. = 2, $p = 0.46$) and no significant effect of time on workers' level of ovarian activation (LM: $\chi^2 = 0.55$, d.f. = 2, $p = 0.76$), showing that differences between F+ and F- workers in levels of ovarian activation were consistent over time.

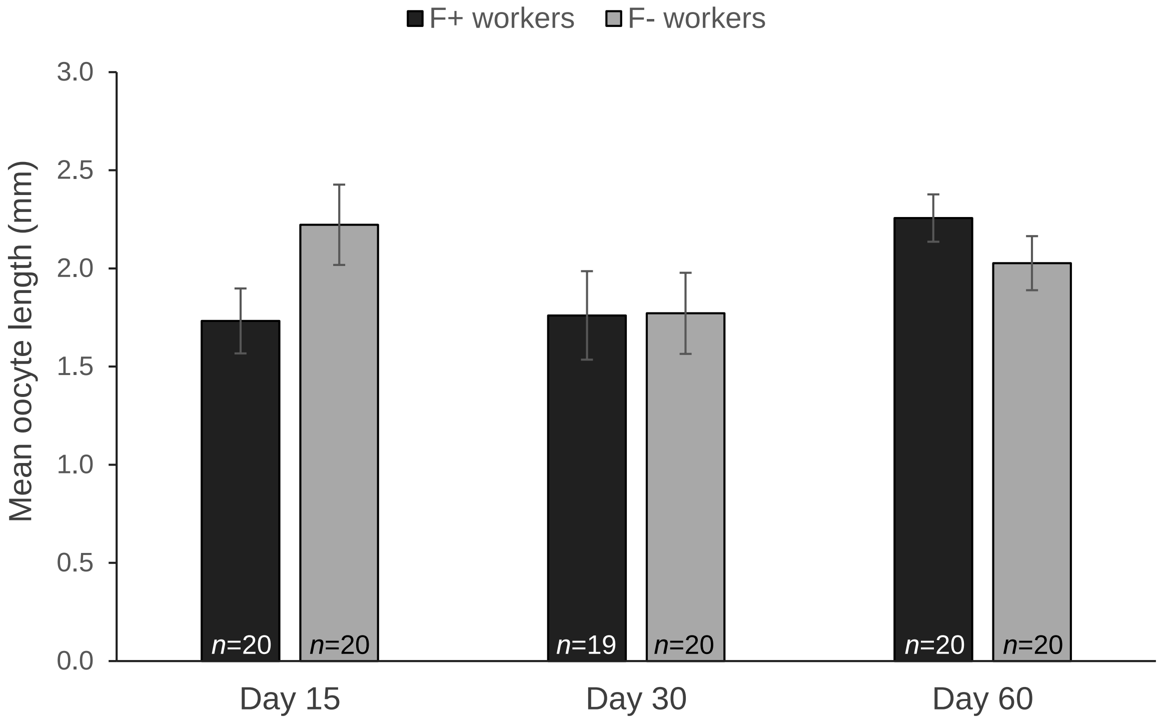

Figure S3. Levels of ovarian activation in non-focal workers from the groups set aside for confirmation of workers' level of ovarian activation (Experiment 2). F+ (black bars) and F- (grey bars) workers were collected at three different time steps (day 15, day 30 and day 60 after group establishment). Level of ovarian activation was measured as the mean length of the eight terminal oocytes. Error bars are ± 1 SE and sample sizes (worker numbers) are inset within the base of each bar. There was no significant interaction between treatment and time (LMM: $\chi^2 = 4.29$, d.f. = 2, $p = 0.12$) and no significant effect of time on workers' level of ovarian activation (LMM: $\chi^2 = 4.69$, d.f. = 2, $p = 0.096$).

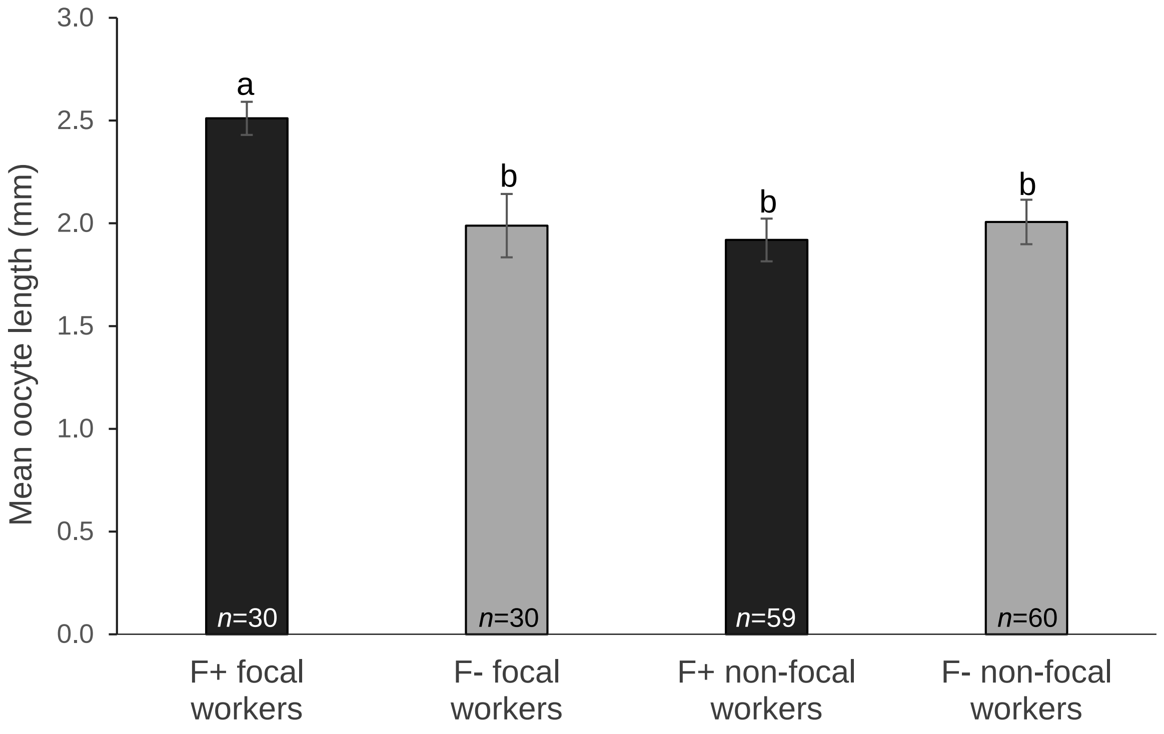

Figure S4. Levels of ovarian activation in all workers (F+, black bars; F-, grey bars) from the groups set aside for confirmation of workers' level of ovarian activation (Experiment 2). Level of ovarian activation was measured as the mean length of the eight terminal oocytes. Data are those in Figures S2 and S3, pooled across all three time periods. The different letters denote statistical differences after post-hoc tests corrected for multiple comparisons with the sequential Bonferroni-Holm procedure. Error bars are ± 1 SE and sample sizes (worker numbers) are inset within the base of each bar.

3. References Cited in Electronic Supplementary Material

1. Alford DV. 1975 *Bumblebees*. London, Davis-Poynter.
2. Owen RE. 1989 Differential size variation of male and female bumblebees. *J. Heredity* **80**, 39-43.
3. Duchateau MJ, Velthuis HHW. 1988 Development and reproductive strategies in *Bombus terrestris* colonies. *Behaviour* **107**, 186-207.
